# Association of a novel 27-gene immuno-oncology assay with efficacy of immune checkpoint inhibitors in advanced non-small cell lung cancer

**DOI:** 10.1186/s12885-022-09470-y

**Published:** 2022-04-14

**Authors:** Harsha Ranganath, Amit L. Jain, Justin R. Smith, Julie Ryder, Amina Chaudry, Emily Miller, Felicia Hare, Poojitha Valasareddy, Robert S. Seitz, David R. Hout, Matthew G. Varga, Brock L. Schweitzer, Tyler J. Nielsen, Janice Mullins, Douglas T. Ross, David R. Gandara, Gregory A. Vidal

**Affiliations:** 1 Division of Hematology/Oncology, University of Tennessee Health Sciences Center, Memphis, TN USA; 2 West Cancer Center and Research institute, 7945 Wolf River Blvd, Germantown, TN 38138 USA; 3 Oncocyte Corporation, Nashville, TN USA; 4 Department of Internal Medicine, UC Davis Medical Center, Sacramento, CA USA

**Keywords:** Tumor biomarkers, Immunotherapy, Gene expression profiling, Programmed death ligand 1, Tumor microenvironment

## Abstract

**Background:**

Immune checkpoint inhibitor (ICI) therapies represent a major advance in treating a variety of advanced-stage malignancies. Nevertheless, only a subset of patients benefit, even when selected based on approved biomarkers such as PD-L1 and tumor mutational burden. New biomarkers are needed to maximize the therapeutic ratio of these therapies.

**Methods:**

In this retrospective cohort, we assessed a 27-gene RT-qPCR immuno-oncology (IO) gene expression assay of the tumor immune microenvironment and determined its association with the efficacy of ICI therapy in 67 advanced-stage NSCLC patients. The 27-gene IO test score (IO score), programmed cell death ligand 1 immunohistochemistry tumor proportion score (PD-L1 TPS), and tumor mutational burden (TMB) were analyzed as continuous variables for response and as binary variables for one-year progression free survival. The threshold for the IO score was prospectively set based upon a previously described training cohort. Prognostic implications of the IO score were evaluated in a separate cohort of 104 advanced-stage NSCLC patients from The Cancer Genome Atlas (TCGA) who received non-ICI therapy.

**Results:**

The IO score was significantly different between responders or non-responders (*p* = 0.007) and associated with progression-free survival (*p =* 0.001). Bivariate analysis established that the IO score was independent of PD-L1 TPS and TMB in identifying patients benefiting from ICI therapy. In a separate cohort of late-stage NSCLC patients from TCGA, the IO score was not prognostic of outcome from non-ICI-treated patients.

**Conclusions:**

This study is the first application of this 27-gene IO RT-qPCR assay in a clinical cohort with outcome data. IO scores were significantly associated with response to ICI therapy and prolonged progression-free survival. Together, these data suggest the IO score should be further studied to define its role in informing clinical decision-making for ICI treatment in NSCLC.

**Supplementary Information:**

The online version contains supplementary material available at 10.1186/s12885-022-09470-y.

## Background

Since the FDA approved the first immune checkpoint inhibitor (ICI) in 2011 to treat late-stage, metastatic melanoma, ICIs have been successfully applied across various cancers [1–3]. In particular, ICIs targeting programmed cell death protein 1 (PD-1) and program cell death ligand 1 (PD-L1) are among the most promising regimens for the treatment of advanced non-small cell lung cancer (NSCLC), as demonstrated by increased progression-free survival (PFS), overall survival (OS), and regulatory approval [4–8]. Although ICI therapies, either alone or combined with other treatments, constitute a significant advance, a substantial proportion do not benefit from treatment [9]. In addition, ICI therapies are costly and are associated with the risk of immune-related adverse events [10, 11]. Although predictive biomarkers such as PD-L1 immunohistochemistry tumor proportion score (PD-L1 TPS) and tumor mutational burden (TMB) are of value in some patients, each suffers from several limitations that compromise their predictive accuracy and use in clinical practice. A variety of different PD-L1 tests have been employed clinically, each with its own individual scoring criteria and all are intrinsically subjective due to spatial and temporal heterogeneity within the tumor immune microenvironment (TIME) [12]. Similarly, multiple techniques have been used to estimate TMB, with variable success in predicting the efficacy of ICIs across different trials [13]. Hence, there is an ongoing unmet need for improved predictive biomarkers of ICI therapy efficacy. The 27-gene IO algorithm and resulting IO score have their origins in the findings of TNBCtype, an unsupervised k-means cluster classification of triple-negative breast cancer (TNBC) [14–16]. TNBCtype classifies TNBC tumors into four tumor subtypes, one of which is the mesenchymal (M) signature, reflecting the epithelial to mesenchymal transition (EMT), and is often associated with exclusion of inflammatory cells [14, 15]. In addition to the tumor subtypes, the TNBCtype classifier also includes an inflammatory cell derived signature termed the immunomodulatory component (IM) and a mesenchymal stem-like (MSL) gene expression signature attributed to cancer associated fibroblasts [14–18]. Given that non-tumor cell types largely contribute to the IM and MSL signatures, and EMT is a commonly found across epithelial tumor types, we hypothesized that combining these signatures would represent a TIME phenotypic classifier applicable to multiple solid tumor types [15, 19]. The IO score was developed to combine the immunomodulatory signature with the EMT-derived and stromal signatures, which contrast checkpoint immuno-responsiveness with immuno-resistance features, respectively [20]. Although initially derived using whole transcriptome methods such as NGS and microarray, the algorithm was translated into an RT-qPCR assay to better meet the need of clinical adaptability. In this proof of principle study, we assessed whether the IO score was significantly associated with ICI therapeutic efficacy among advanced-stage NSCLC patients and conversely, whether it was specific to ICIs or more generally prognostic of outcome in patients treated by cytotoxic chemotherapy alone.

## Materials and methods

### Study cohort

In this retrospective community cohort study, we obtained archival formalin-fixed paraffin embedded (FFPE) tumor tissue from 67 advanced-stage NSCLC (recurrent stage II or later) patients treated with one of three ICIs (pembrolizumab, nivolumab, or atezolizumab) prescribed either as a single agent (57 patients) or in combination with cytotoxic chemotherapeutic drugs pemetrexed and carboplatin (ICI + Chemo, *n* = 10 patients) from whom efficacy data were available from the West Clinic Cancer Center and Research Institute (Germantown, TN). Patients were selected for the study by retrospective chart review to identify those who initiated ICI treatment between April, 2015 and May, 2018 with sufficient follow-up records and biopsy material for testing. This study was performed in accordance with the Declaration of Helsinki and approved by the University of Tennessee Health Science Center Institutional Review Board (18–05806-xp). Informed consent was obtained from all subjects and or their legal guardians.

Response was defined using RECIST 1.1 criteria as responders or non-responders (stable disease or progressive disease) [21]. For this study, patients were censored to include only those who had at least 8 weeks of clinical follow-up post-treatment without an event.

### PD-L1 TPS and TMB

Data on PD-L1 TPS and TMB were available in the clinical records for a subset of patients. The PD-L1 immunohistochemistry and TMB testing were performed by Caris Life Sciences as described previously [22]. In brief, slides of FFPE tissue from tumor samples were stained using the antibody 22c3 (Dako, Santa Clara, CA, USA) and PD-L1 TPS was defined as positive or negative by a threshold of ≥1% as described in KEYNOTE-042 [23]. PD-L1 TPS was measured as a continuous variable for objective response analysis. Six patients with PD-L1 results we reported only as “positive” or “negative” (as defined by ≥1%) and therefore could not be assessed on a continuous scale. TMB was performed by next generation sequencing and calculated by determining the number of nonsynonymous somatic mutations excluding any known single nucleotide polymorphisms found in dbSNP (version 137) or the 1000 Genomes Project database (phase 3; http://www.internationalgenome.org**/**). TMB high was defined as ≥10 mutations per megabase (mut/MB), determined by Caris Life Sciences [22]. TMB was also assessed as a continuous variable for objective response.

### Derivation of IO score

Design of the 27-gene IO algorithm and resulting IO score was guided by findings from Lehmann *et. al*., which suggested that the gene expression patterns for the IM and MSL components of the TNBCtype model were descriptive of the tumor microenvironment, and findings from the 101-gene TNBCtype model by Ring *et. al*. which identified the IM signature to be a modifier of TNBC tumor subtypes [15, 16]. Further analysis of the TNBCtype model also found a strong inverse relationship between the M and IM subtypes. Together, this led to the biological hypothesis that inclusion of the IM, M, and MSL features into a single algorithm could yield the most information regarding the immunogenic state of the TIME [17]. To create a test more adapted to the clinical setting, a reduced set of 27 genes that best identified the IM, M and MSL classes were selected from the 101-gene TNBCtype model and translated to an RT-qPCR assay. No datasets that included patients treated with ICI were used in the training of the 27-gene IO algorithm. Despite implementing a new platform to measure expression, the threshold for positivity remained set at an IO score of ≥0.09 as initially described [17]. Input for the IO score is obtained through Ct values resulting from the RT-qPCR panel containing the requisite 27-genes [24].

To perform the RT-qPCR panel, RNA was purified from FFPE tissue using the QIAGEN RNeasy FFPE Kit, according to the kit protocol. A minimum concentration of 3.57 ng/μL of RNA, as measured by fluorometric quantification (Qubit 2.0, Thermo Fisher Scientific, Waltham, Massachusetts, USA), is needed to meet the input requirement of 50 ng. Up to 14 μL of RNA, totaling 50 ng, was used for the SuperScript VILO cDNA Synthesis Kit (Thermo Fisher Scientific, Waltham, Massachusetts, USA) in a 20 μL volume reaction. Following cDNA synthesis, 2.5 μL of cDNA was added to 7.5 μL of TaqMan PreAmp Master Mix (Thermo Fisher Scientific, Waltham, Massachusetts, USA) plus a gene-specific primer pool for a total volume of 10 μL. The preamplification reaction was performed for 14 cycles according to the manufacturer’s recommendations. The preamplification was diluted 1:20 in TE buffer, and 3 μL of the diluted preamplification product was distributed as template for the 27-gene IO test which was spotted in a 384-well plate using the TaqMan Multiplex Master Mix with a 10 μL final volume and cycled on the QuantStudio 6 as follows: activation at 95 °C for 20 s; followed by alternating denaturation at 95 °C for 1 s and extension at 60 °C for 20 s for a total of 40 cycles. Results were exported, quality control metrics were assessed, and data were processed by the 27-gene IO algorithm to determine the IO score of each sample.

### The Cancer Genome Atlas (TCGA) prognostic assessment

A prognostic assessment was performed as an exploratory endpoint of overall survival by downloading RNA expression data generated by the TCGA Research Network (https://www.cancer.gov/tcga) from the LUAD and LUSQ data sets containing 848 patients (accessed May 2, 2020). We then selected those at stage 3 or above who received chemotherapy, yielding a total of 104 patients for our analysis. Overall survival at 2 years was estimated and Cox proportional hazards ratio was determined with 95% CIs.

### Statistical analyses

Statistical analyses were performed using R version 4.0.3 (https://cran.r-project.org) software. PFS up to 1 year was the predefined primary endpoint and objective response to ICI therapy was the secondary endpoint in this prospectively defined retrospective study. The IO score, PD-L1 TPS and TMB were measured as continuous variables between responders (CR, PR) and non-responders (SD, PD). Comparisons between groups for each biomarker were then conducted using Welch’s t-test. Demographic data, clinical characteristics, and the three biomarkers (IO score, PD-L1 TPS, and TMB) were analyzed as categorical variables and evaluated for effect by performing univariate cox proportional hazards regression models with 95% confidence intervals (CIs). The IO score was paired with either demographic factors, clinical attributes, PD-L1 TPS, or TMB to generate bivariate Cox proportional hazard ratios. Kaplan-Meier curves were plotted to estimate survival within the three biomarker variables. Survival analysis were measured for significance by the log-rank test.

## Results

### Patient characteristics

A total of 67 metastatic NSCLC patients were analyzed (Table 1). Of these, 57 (85%) patients received ICI monotherapy and 10 (15%) patients received ICI plus chemotherapeutic agents pemetrexed and carboplatin (ICI + Chemo). ICI therapy consisted of nivolumab (49%), pembrolizumab (48%), or atezolizumab (1%). A total of 42 (63%) patients were responders and 25 (37%) patients were non-responders. IO scores were assessabled from biopsy samples for all 67 patients (45% primary, 55%, metastatic). Because this is a retrospective study, PD-L1 TPS data were available for only 59 patients (ICI + Chemo *n* = 9) and TMB data were available for only 36 of the 67 patients (ICI + Chemo *n* = 8) in the study population. Univariate analyses showed no significant association between one-year PFS and either age, sex, race, histology, biopsy site, ECOG, or ICI therapy (Supplemental Fig. S1).

**Table 1:** Patient characteristics

Characteristic	Patients, *n* (%)
Age at IO Therapy (years)	
≤ 50	5 (8%)
51–60	14 (21%)
61–70	21 (31%)
> 70	27 (40%)
Sex	
Female	31 (46%)
Male	36 (54%)
Race	
African American	20 (30%)
Caucasian	47 (70%)
Disease Stage	
Stage 2^a^	2 (3%)
Stage 3	9 (13%)
Stage 4	56 (84%)
Histology	
Adenocarcinoma	36 (54%)
Squamous	17 (25%)
Adenosquamous	2 (3%)
NOS	12 (18%)
Biopsy Site	
Primary carcinoma	30 (45%)
Distant metastases	37 (55%)
ECOG performance status	
0	23 (34%)
1	30 (45%)
2	14 (21%)
Type of Therapy	
ICI Monotherapy	57 (85%)
ICI + Chemotherapy	10 (15%)
ICI therapy received	
Nivolumab	33 (49%)
Pembrolizumab	32 (48%)
Nivolumab/ Pembrolizumab	1 (1%)
Atezolizumab	1 (1%)
Line of Therapy	
1st line	29 (43%)
2nd line +	38 (57%)
Response Status	
Response (CR, PR)	42 (63%)
Non-Response (PD, SD)	25 (37%)
IO Score (≥0.09)	
Positive, *n* (%)	36 (54%)
Negative, *n* (%)	31 (46%)
PD-L1 TPS (≥1%)	
Positive, *n* (%)	45 (67%)
Negative, *n* (%)	14 (21%)
Missing, *n* (%)	8 (12%)
TMB (≥10 mut/MB)	
Positive, *n* (%)	21 (31%)
Negative, *n* (%)	15 (22%)
Missing, *n* (%)	31 (46%)

*NOS* not otherwise specified, *ICI* immune checkpoint inhibitor, *IO score* immuno-oncology score, *NSCLC* non-small cell lung cancer, *PD-L1 TPS* PD-L1 immunohistochemistry tumor proportion score, *TMB* tumor mutational burden

^a^Recurrent Disease

### Comparisons of biomarker values between responders and non-responders

**Fig. 1:**
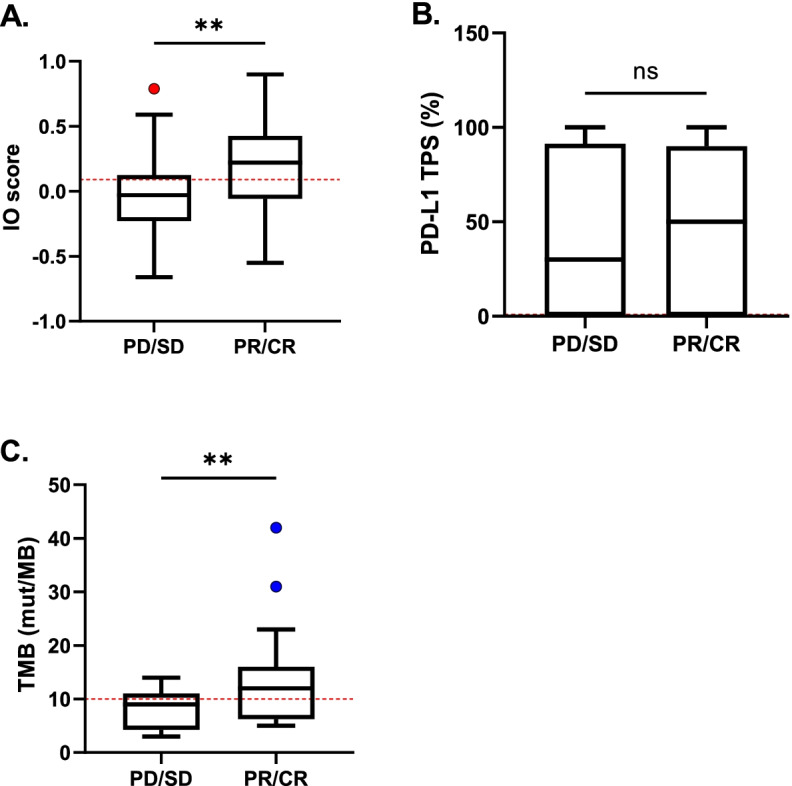
Comparisons between biomarker measurements and response to ICI therapy. **A-C** IO score (*n* = 67), PD-L1 TPS (*n* = 53), and TMB (*n* = 36) were plotted as a continuous variable by responders (CR, PR) and non-responders (PD, SD). Box and whisker plots are shown. For reference, the positivity thresholds are indicated as dashed red lines at ≥0.09 for IO score, ≥1% for PD-L1 TPS, and ≥ 10 mut/MB for TMB. PD, progressive disease; SD, stable disease; PR, partial response; CR, complete response. Comparisons between groups were conducted using Welch’s t-test. **p < 0.01

To compare response for each of the three biomarkers, we plotted the continuous biomarker value (IO score, PD-L1 TPS, and TMB) in the responder and non-responder groups who had received either ICI monotherapy or ICI with chemotherapy (Fig. 1). The IO score and TMB value showed significant differences between the responder and non-responder groups (*p* < 0.01 for both biomarkers) whereas the PD-L1 IHC TPS displayed no significant difference between groups. When comparing objective response rates, IO positive patients had a rate of 78% (28 out of 36 patients), compared to 64% (29 out of 45) for PD-L1 ≥ 1 and 76% (16 out of 21) for TMB ≥ 10 (Supplemental Table S1). Together, these data suggest that, similar to TMB, the IO score can differentiate responders and non-responders to ICI therapies.

### Biomarker positivity and one-year PFS

**Fig. 2:**
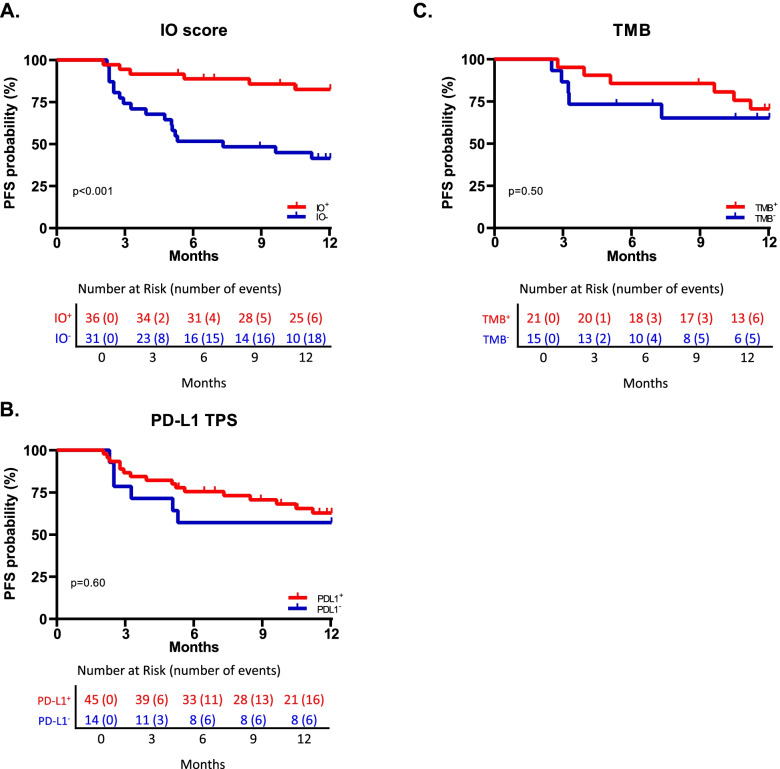
Kaplan-Meier curves showing the association between biomarkers and one-year PFS. **A** IO score stratified by the prospectively set ≥0.09 positivity threshold for 67 NSCLC patients; **B** PD-L1 TPS stratified by the 1% positivity threshold for 59 NSCLC patients; **C** TMB stratified by the ≥10 mut/MB threshold for 36 NSCLC patients. **A-C** significance values calculated by log-rank test

For all patients who had received either ICI monotherapy or ICI + Chemo, Kaplan-Meier curves were plotted using up to one-year PFS against each biomarker with positivity thresholds of ≥0.09 for the IO score (*n* = 67), ≥1% for PD-L1 TPS (*n* = 59), and ≥ 10 mut/MB for TMB (*n* = 36) (Fig. 2). Univariate Cox proportional hazards analysis showed that the IO score was significantly associated with up to one-year PFS, yielding a hazard ratio of 0.21 (95%CI 0.085–0.54, *p* < 0.001) (Fig. 2A). In contrast, no significant associations were found for PD-L1 TPS (hazard ratio 0.76, 95%CI 0.30–1.93; *p* = 0.60) (Fig. 2B) or for TMB (hazard ratio 0.69, 95%CI 0.21–2.27; *p* = 0.50) (Fig. 2C). The IO score remained significant when comparing median PFS and 1-year overall survival (*p* = 0.025 and *p* = 0.01 respectively, Supplemental Fig. S2). Furthermore, the IO score was significantly associated with up to one-year PFS when evaluated in the subset of patients for whom PD-L1 TPS data were available (hazard ratio 0.24, 95%CI 0.093–0.61, *p* = 0.001, *n* = 59) and for whom TMB data were available (hazard ratio 0.18, 95%CI 0.38–0.82, *p* = 0.01, *n* = 36) (Supplemental Fig. S3). The IO score was next examined in univariate and bivariate analyses with patient demographics, where no single factor was significantly associated with up to one-year PFS by Cox proportional hazards (Supplemental Fig. S1B). Furthermore, bivariate analysis showed that the IO score was independent of whether the patient had been treated with either pembrolizumab or nivolumab (hazard ratio 0.23, 95%CI 0.090–0.57, *p* = 0.002, *n* = 65), independent of either adenocarcinoma or squamous histology (hazard ratio 0.17, 95%CI 0.05–0.52, *p* = 0.002, *n* = 53), and independent of primary or metastatic biopsy site, (hazard ratio 0.21, 95%CI 0.084–0.54, *n* = 67).

### IO score and up to one-year PFS in patients receiving ICI monotherapy

**Fig. 3:**
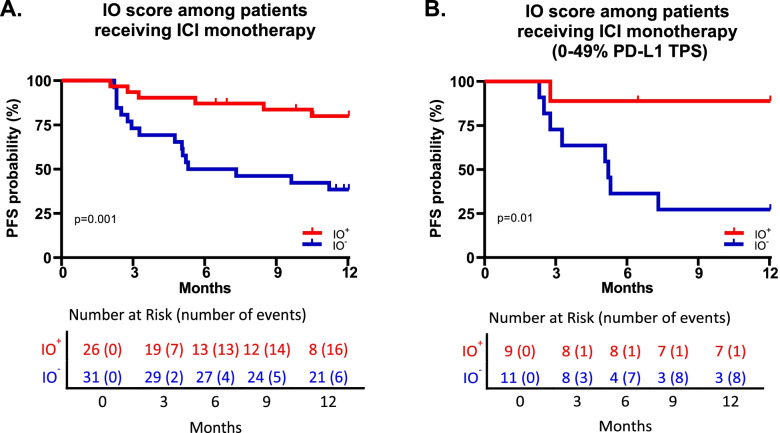
Kaplan-Meier curves showing the association between IO score and one-year PFS in patients receiving ICI monotherapy with either (**A**) all patients who received ICI monotherapy (*n* = 57) or (**B**) negative or low PD-L1 TPS (0–49%, *n* = 20). Significance values calculated by log-rank test. PFS, progression-free survival

To determine if the IO score could better inform clinical decisions for treatment of patients with negative or low PD-L1 TPS where cytotoxic chemotherapy may be used in combination with ICIs, we assessed the association of IO scores with up to one-year PFS among patients who received ICI as monotherapy versus combined with cytotoxic chemotherapy. In the 57 patients who received ICI monotherapy, univariate Cox proportional hazards analysis showed that the IO score was significantly associated with up to one-year PFS, yielding a hazard ratio of 0.23 (95%CI 0.09–0.60, *p* = 0.001, *n* = 57) (Fig. 3A). Twenty of the patients who received ICI monotherapy were either PD-L1 TPS negative or were positive below the 50% TPS level (0%, *n* = 11; 1–5%, *n* = 5; 20%, *n* = 1; 25%, *n* = 1; and 30%, *n* = 2). Nine of these twenty (45%) low PD-L1 expressors treated with monotherapy were positive for IO score, and the IO score was significantly associated with one-year PFS (hazard ratio 0.11, 95%CI 0.013–0.87, *p* = 0.01, *n* = 20) (Fig. 3B).

### IO score is not prognostic for chemotherapy effect in advanced-stage NSCLC patients from TCGA

**Fig. 4:**
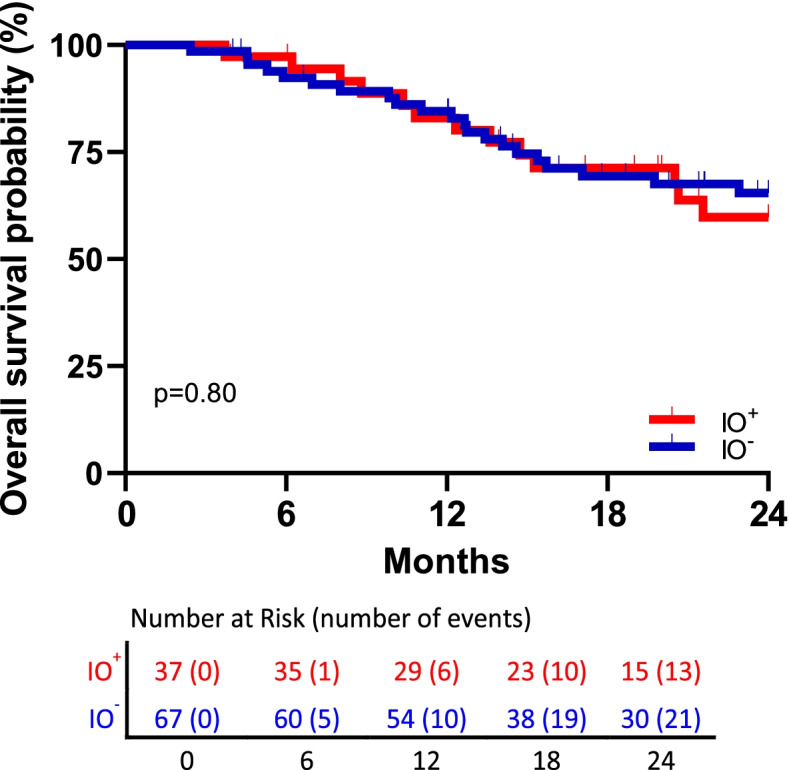
Kaplan-Meier curves showing 2-year overall survival in 104 advanced stage NSCLC patients treated with non-ICI therapy from TCGA in relation to the IO score (log-rank test, *p* = 0.80)

We next sought to examine prognostic inference of the IO score in a separate cohort of late-stage NSCLC patients from the TCGA (*n* = 104). In this cohort of patients who had not received ICI therapy, we assessed IO score positivity and 2-year overall survival (Fig. 4). Univariate analysis of these data indicate that the IO score is not prognostic for 2-year overall survival in late-stage NSCLC patients who were treated with cytotoxic chemotherapy (hazard ratio 1.11, 95%CI 0.56–2.23, *p* = 0.80).

## Discussion

While ICIs have demonstrated tremendous promise, when applied in all-comer populations ICIs improve clinical outcomes in only a subset of patients and may even cause life-threatening or fatal immune-related adverse events in some individuals [9–11]. Thus, there remains a need for new and better biomarkers to discern patients who will likely benefit from these therapies. Recently, we have described the derivation of a 27-gene IO algorithm obtained from analysis of whole transcriptome expression data of TNBC patients to address this unmet need [17]. The algorithm was built around the hypothesis that by assessing the immunomodulatory (IM), mesenchymal (M) and mesenchymal stem like (MSL) features of the TIME, the changes in gene expression patterns would translate to multiple tissues and provide greater adaptability for clinical application [20].

To adapt the algorithm for broader clinical usage, the IO score was translated to an RT-qPCR panel while still utilizing the predefined threshold obtained through transcriptome analysis. As the patients used in the development of the assay were ICI naive, the thresholds were trained and defined only by the TIME phenotypic classifications. The implementation of the RT-qPCR panel greatly reduces the sample input requirements and the turnaround time for actionable results. Furthermore, the use of a predefined binary threshold to separate patients into two diagnostic categories enhances clinical utility where treatment choices are binary [24].

The results described herein demonstrate that this TIME phenotypic classifier, trained upon TNBC tumors, has a strong association with ICI efficacy in NSCLC. To further investigate clinical adaptability of the IO score, exploratory bivariate analyses demonstrated independence from ICI used and from squamous cell or adenocarcinoma histology (Supplemental Fig. S1). Recent reports have shown that TMB high (≥10 mut/Mb) fails to predict improved objective response or overall survival to ICI therapies in several tumor types, especially those with squamous histology [25]. These data suggest that the IO score and TMB might bring independent information to ICI efficacy classification and warrants further prospective study. Given the possibility that the TIME between primary and metastatic sites could be different enough to influence the performance of the IO score, we tested for and found independence from the site of the biopsy (Supplemental Fig. S1). This result, if verified in subsequent studies, may have the practical implication of increasing access to patients from whom a primary tumor biopsy may be unavailable.

The results reported in this study may reflect the IO score’s unique measurement of a combination of the IM, MSL, and M components of the TIME. While these subtypes of TNBC have been noted since 2011, these three components reflect common biology across multiple tumor types including TNBC and NSCLC [14, 17]. Utilization of all three components in the IO score may explain its apparent plasticity, allowing for the measurement of factors predicting response to ICIs across multiple tumor types, as they include stroma (MSL), the tumor (M), and the inflammatory cells (IM) [20, 26]. Furthermore, an advantage of the IO score is that the IM component corresponds to the positive signal, associated with sensitivity to ICIs, while the M and MSL components deliver a negative signal, associated with resistance. The relative abundance of these opposing signals may offer some redundancy in phenotypic classification, serving to reduce the requirement of relatively high tumor content and reducing the impact of stochastic sampling differences.

The idea of an immune checkpoint predictive biomarker that contains both positive and negative components is not unique to the IO score [19]. However, it is novel as most gene expression algorithms, along with commercially available immune gene expression panels, tend to focus only on measuring the inflammatory state of the TIME and use this inflammatory state as a surrogate for the ability of the immune system to act against the tumor cells if the “checkpoint” is inhibited. In addition to the practical advantages of having positive and negative signaling for setting a reproducible threshold, examining alternate states of the TIME could likewise be a surrogate for the lack of immune response against the tumor. Further, we hypothesize inclusion of a dichotomic signal will increase the predictive nature of this biomarker as opposed to similar biomarkers which are limited as prognostic tools. This hypothesis is indirectly supported by the observation that IO score was not significant with 2-year overall survival when assessing available data from TCGA for chemotherapy, suggesting the IO score is not broadly prognostic and is only informative in the presence of an ICI.

We fully recognize the strengths and limitations of this analysis. Strengths include establishing a prospectively defined endpoint for this retrospective study, blinded assessment of the IO score, comparison to other approved biomarkers such as PD-L1 and TMB, demonstrating independence from therapeutic, demographic, and histological factors, the establishment of a predetermined threshold for binary assessment of efficacy and using the more clinically applicable RT-qPCR version of the IO score. Limitations consist of the retrospective nature of the analysis, the limited patient sample size, dependence on a real-world approach to patient data availability, and potential unknown biases in favorable characteristics of patients selected for ICI therapy.

## Conclusions

The IO score is driven by a balanced approach of assessing three components of the TIME (IM, MSL, M), which may explain its translatability from TNBC to NSCLC. Moreover, the IO score provides information that is independent of PD-L1 TPS and TMB and is not simply a prognostic indicator. As a measure of the TIME and the mesenchymal features of the tumor, the IO score may help better inform clinical decisions for ICI treatment of cancer patients. Thus, these results warrant further study of the IO score.

## Supplementary Information

**Additional file 1: Supplemental Figure S1.** Forest plots of hazard ratios for (A) patient demographics and for (B) IO score alone and in combination with patient demographics as defined in Table 1. **Supplemental Figure S2.** Kaplan-Meier plots of median progression free survival and 1-year overall survival by (A, B) IO score (*n* = 67), (C, D) PD-L1 IHC TPS (*n* = 56), and (E, F) TMB (*n* = 36). **Supplemental Figure S3.** Kaplan-Meier curves showing the association between biomarkers and one-year PFS. (A) IO score and PD-L1 TPS for 62 NSCLC patients; (B) IO score and TMB for 36 NSCLC patients. Significance values calculated by log-rank test. **Supplemental Table S1.** Test Characteristics for each biomarker with available data in this cohort.

## Data Availability

The data sets generated and analyzed during the current study are not publicly available due to the full clinical files from patients in this manuscript are confidential information from a private clinic but are available from the corresponding author upon reasonable request. The data from TCGA are publicly available.
