# Supplementary material for: Association of a novel 27-gene immuno-oncology assay with efficacy of immune checkpoint inhibitors in advanced non-small cell lung cancer
Source: BMC Cancer. 2022 Apr 14;22:407. doi: 10.1186/s12885-022-09470-y (PMC9008990; doi:10.1186/s12885-022-09470-y)
Supplement: Supplementary file 1 — Additional file 1: Supplemental Figure S1. Forest plots of hazard ratios for (A) patient demographics and for (B) IO score alone and in combination with patient demographics as defined in Table 1. Supplemental Figure S2. Kaplan-Meier plots of median progression free survival and 1-year overall survival by (A, B) IO score (n = 67), (C, D) PD-L1 IHC TPS (n = 56), and (E, F) TMB (n = 36). Supplemental Figure S3. Kaplan-Meier curves showing the association between biomarkers and one-year PFS. (A) IO score and PD-L1 TPS for 62 NSCLC patients; (B) IO score and TMB for 36 NSCLC patients. Significance values calculated by log-rank test. Supplemental Table S1. Test Characteristics for each biomarker with available data in this cohort. [file 12885_2022_9470_MOESM1_ESM.docx]

**Supplemental Figure S1**. Forest plots of hazard ratios for (A) patient demographics and for (B) IO score alone and in combination with patient demographics as defined in Table 1.

**Supplemental Figure S2.** Kaplan-Meier plots of median progression free survival and 1-year overall survival by (A, B) IO score (n=67), (C, D) PD-L1 IHC TPS (n=56), and (E, F) TMB (n=36).


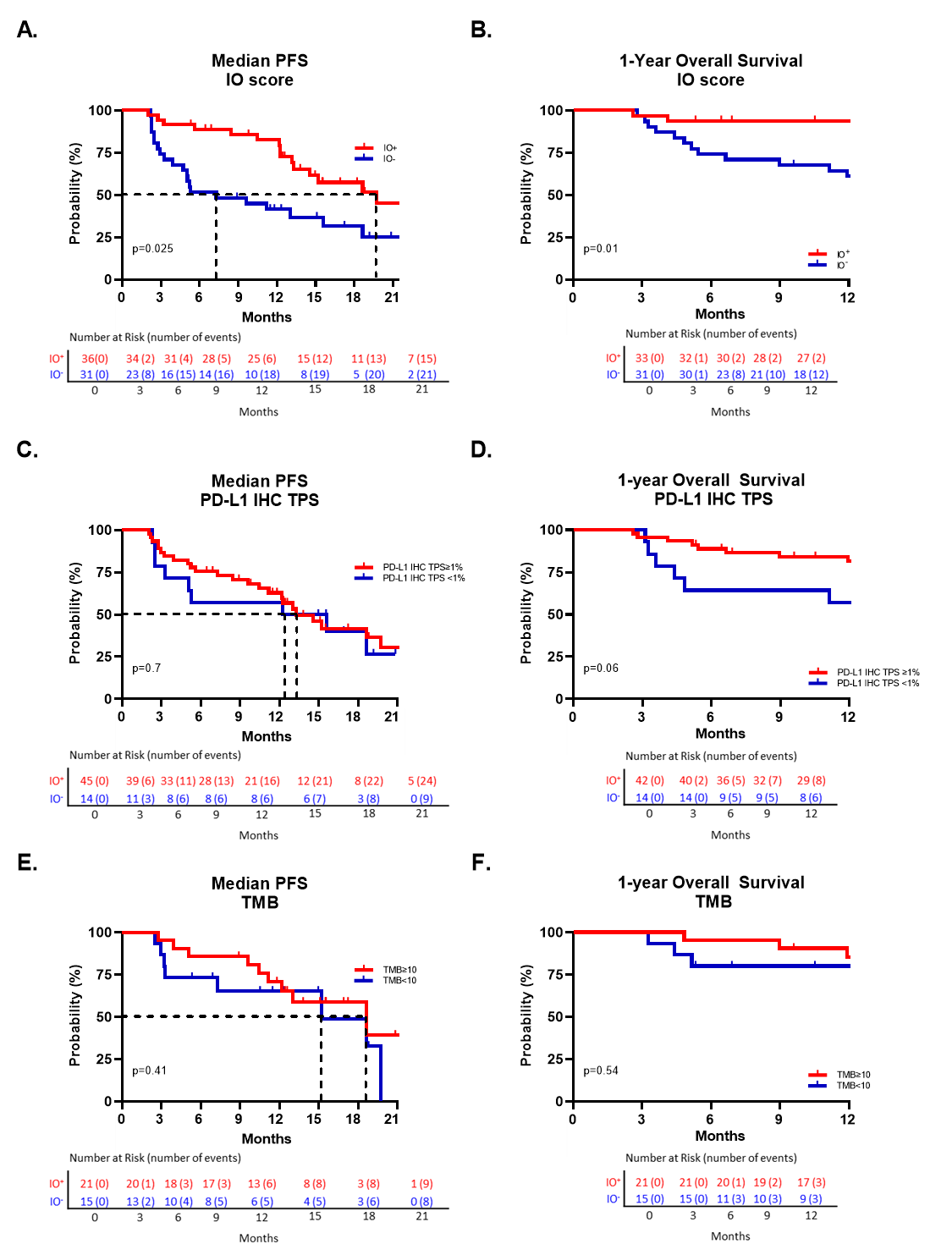


**Supplemental Figure S3:** Kaplan-Meier curves showing the association between biomarkers and one-year PFS. (A) IO score and PD-L1 TPS for 62 NSCLC patients; (B) IO score and TMB for 36 NSCLC patients. Significance values calculated by log-rank test.

**Supplemental Table S1**

Test Characteristics for each biomarker with available data in this cohort.

| Objective Response Rate (ORR) by Biomarker | | | |
| --- | --- | --- | --- |
|  | IO Score + | PD-L1 (≥1%) | TMB (≥10) |
| Non-responders | 8 | 16 | 5 |
| Responders | 28 | 29 | 16 |
| ORR | 77.8% | 64.4% | 76.2% |
